# The recovery after Achilles tendon rupture: a protocol for a multicenter prospective cohort study

**DOI:** 10.1186/s12891-019-2437-z

**Published:** 2019-02-11

**Authors:** Olivier C. Dams, Inge van den Akker-Scheek, Ron L. Diercks, Klaus W. Wendt, Eelke Bosma, Tom M. van Raaij, Arvid V. Munzebrock, Wierd P. Zijlstra, Johannes Zwerver, Inge H. F. Reininga

**Affiliations:** 1 Department of Sport and Exercise Medicine, University of Groningen, University Medical Center Groningen, Groningen, The Netherlands; 2 Department of Orthopedics, University of Groningen, University Medical Center Groningen, Groningen, The Netherlands; 3 Department of Trauma Surgery, University of Groningen, University Medical Center Groningen, Groningen, The Netherlands; 4 Department of Surgery, Martini Hospital, Groningen, The Netherlands; 5 Department of Orthopaedic Surgery, Martini Hospital, Groningen, The Netherlands; 6 Department of Surgery, Medical Center Leeuwarden, Leeuwarden, The Netherlands; 7 Department of Orthopaedic Surgery, Medical Center Leeuwarden, Leeuwarden, The Netherlands

**Keywords:** Achilles tendon rupture, PROM, Ultrasound tissue characterization, Prospective cohort, Multicenter, Rehabilitation, Economic, Shared-decision making

## Abstract

**Background:**

Achilles tendon rupture (ATR) is a common sports injury, with a rising incidence and significant impairments. Due to the lack of treatment guidelines, there is no consensus about diagnostic methods, primary treatment (non-surgical or surgical) and rehabilitation. It is hypothesized that this lack of consensus and guidelines leads to sub-optimal recovery and higher societal costs.

The primary aim of this study is to give a broad insight into the recovery after ATR. Secondarily this study aims to explore factors contributing to recovery and gain insight into the cost-effectiveness of ATR management.

**Methods:**

This multicenter prospective cohort study will include all adult (≥ 18 years) patients with an ATR treated at the three main hospitals in the Northern Netherlands: University Medical Center Groningen, Martini Hospital Groningen and Medical Center Leeuwarden. All subjects will be invited for three visits at 3, 6 and 12 months post-injury. The following data will be collected: patient-reported outcome measures (PROMs), physical tests, imaging and economic questionnaires. At 3 months post-injury personal, injury, and treatment data will be collected through a baseline questionnaire and assessment of the medical file. The PROMs concern the Dutch version of the Achilles Tendon Total Rupture Score, EQ-5D-5 L, Oslo Sport Trauma Research Center Overuse Injury Questionnaire, Injury Psychological Readiness Return to Sport Scale, Tampa Scale of Kinesiophobia, Expectations, Motivation and Satisfaction questionnaire and a ranking of reasons for not returning to sport. The administered physical tests are the heel-rise test, standing dorsiflexion range of motion, resting tendon length and single leg hop for distance. Ultrasound Tissue Characterization will be used for imaging. Finally, economic data will be collected using the Productivity Cost Questionnaire and Medical Consumption Questionnaire.

**Discussion:**

This prospective cohort study will contribute to optimal decision making in the primary treatment and rehabilitation of ATRs by providing insight into (1) ATR recovery (2) novel imaging for monitoring recovery (3) (barriers to) return to sport and (4) cost-effectiveness of management. The analysis of these data strives to give a broad insight into the recovery after ATR as well as provide data on novel imaging and costs, contributing to individualized ATR management.

**Trial registration:**

Trialregister.nl. NTR6484. 20/06/2017. 20/07/2017.

## Background

The Achilles tendon is the strongest and thickest tendon in the human body [1, 2]. Despite its size, it is also the most frequently ruptured tendon. Achilles tendon rupture (ATR) usually occurs due to overloading of the tendon, often in a sport setting [2–4]. ATR has an acute presentation of severe pain, inability to bear weight, and weakness [2]; these disabilities can persist for more than 10 years after injury [5–8]. The incidence of ATR is steadily increasing globally [9–13]; this increase is most prominent in the elderly, who are participating in recreational physical activity more often than in the past [14–16].

Despite the high and increasing burden, consensus on ATR management is lacking. The American Academy of Orthopedic Surgeons (AAOS) published the only international guidelines, however these guidelines have a limited or inconclusive recommendation for the role of imaging, the choice of primary treatment, the methods of rehabilitation and the advised time to return to sport (RTS) [17]. Currently, management decisions depend mostly on the experience and perspective of the practitioner who sees the patient first [18]. Surgical and non-surgical treatment are both supported by literature [5] and the rehabilitation starts at 3 months post-injury, as recommended by the AAOS [5, 17].

Because there is conclusive evidence that outcomes after surgical and non-surgical treatment of ATRs are comparable [5], methods of rehabilitation are becoming increasingly significant [19–24]. Despite this, data on the course of the recovery after ATR are still limited, potentially resulting in suboptimal rehabilitation. Specifically, data such as psychosocial factors related to outcome (including return to sport) after ATR treatment as well as novel imaging is lacking and ATR patient continue to be burdened with a high rates of re-rupture and complications [5, 25, 26] and unpredictable recovery and return to sport (RTS) [6, 27–29]. Several patient-related (BMI, comorbidities and athletic status) and injury-related (delay in presentation, injury etiology, gap-size) factors have a possible influence on the recovery and final outcome [26, 30–35]. However, the role of these factors on ATR recovery using multiple, comprehensive outcomes has not been analyzed. Especially because the overall difference in outcome based on primary treatment (surgical or non-surgical) is minimal [5], it is important for clinicians to individualize treatment and make evidence based decisions based on specific patient and injury-related factors.

To reach this, it is essential to enhance knowledge concerning the recovery from the *patient’s perspective* (physical functioning, quality of life), the *clinical perspective* (tendon structure and strength) and *societal perspective* (costs and participation). Hence, this study aims to give a broad insight into the recovery after ATR as well as provide data on novel imaging and costs, thereby providing data that allows clinicians to individualize ATR management.

### Aims

The primary aim of this study is to give a broad insight into the recovery after ATR. Secondarily this study aims to explore factors contributing to recovery and gain insight into the cost-effectiveness of ATR management.

## Methods

### Design

A multicenter prospective cohort study will be conducted. This study has been approved by the Medical Ethical Committee (METc) of the University Medical Center Groningen (UMCG) (METc 2017/126). This study was locally approved (local feasibility) by the medical ethical committees of the Martini Hospital Groningen (MHG) (MEC 2017–087) and Medical Center Leeuwarden (MCL) (COV 274(a)).

### Participants and setting

Eligible patients are all patients with an ATR who are treated at the three largest hospitals in the Northern Netherlands: UMCG, MHG and/or MCL. Patients will be included within the first 3 months post-injury.

*Inclusion criteria:*

- Older than 18 years of age at the time of inclusion
- Clinically diagnosed with an ATR and treated at the UMCG, MHG and/or MCL

*Exclusion criteria:*

- Unable to understand written Dutch
- Physically unable to perform the tests and/or cognitively unable to complete the questionnaires

### Sample size calculation

A formal sample size calculation is difficult due to the exploratory design of this cohort study as well as the lack of comparable data on ATR recovery. All eligible patients within the designated inclusion period who consent to participate will be included. Based on hospital data indicating the treatment of approximately 15 ATRs per hospital per year as well as similar studies by the research group showing a dropout of 10–20%, we estimate 50 patients will be included. This number will allow us to include at least 5 independent variables in the regression analyses based on “the rule of thumb” of 10 subjects per variable (one in ten rule) [36–38]. Other studies assessing ATR recovery usually included lower patient numbers [20, 27, 39, 40].

### Study procedures

#### Recruitment

The conducting researcher (OCD) will screen the list of patients treated at the Emergency Departments of the UMCG, MHG or MCL and contact all who have been treated for an ATR. All subjects will receive oral and written information about the study prior to giving informed consent.

#### Data collection

Upon consent for participation each subject will be invited for three visits: 3, 6 and 12 months post-injury for data collection. This timeframe is chosen based on the recommendations of the AAOS enabling return to work/sport within 3–6 months and the recovery phase of ATR management starting at 3 months post-injury.

### Measurements

Table 1 presents an overview of the specific data collected.

**Table 1:** Measurements per visit

Data Category	Outcome measure/tool^a^	Month 3	Month 6	Month 12
Demographic and Lifestyle	Baseline questionnaire	X		
Medical	Medical file	X	X	X
PROMs	ATRS-NL^b^ questionnaire	X	X	X
	EQ-5D-5 L questionnaire	X	X	X
	OSTRC^c^-Overuse questionnaire	X	X	X
	I-PRRS^d^	X	X	X
	TSK^e^	X	X	X
	Expectations, motivations and satisfaction questionnaire	X	X (only satisfaction and motivation)	X (only satisfaction and motivation)
	Reasons for not RTS		X	X
Physical tests	Heel-rise test	X	X	X
	Ankle dorsiflexion ROM^f^	X	X	X
	Tendon length	X	X	X
	Single leg hop for distance			X
Imaging	UTC^g^	X	X	X
Economic questionnaires	iPCQ^h^	X	X	X
	iMCQ^j^	X	X	X

^a^ A detailed description of the specific outcome measures/tools is given in the subsection measurements. ^b^. Dutch version of the Achilles Tendon Total Rupture Score ^c^. Oslo Sports Trauma Research Center ^d^. Injury Psycological Readiness to Return to Sport ^e^ Tampa Scale of Kinesiophobia ^f^. Range of motion ^g^. Ultrasound Tissue Characterization ^h^. Productivity Cost Questionnaire ^j^. Medical Consumption Questionnaire

#### Demographic and lifestyle data

A baseline questionnaire concerning personal subject data was constructed for specific use in this study. The items in this questionnaire concern biographical information (age), anthropometrics (height, weight), lifestyle factors (smoking, (level of) physical activity, work, urban/rural inhabitant), personal and family medical history including injuries and tendon complaints, injury (etiology, extent, and symptoms) and management factors during rehabilitation (physiotherapy).

#### Medical data

The patients’ medical files will be inspected at baseline for medical history, medication, injury (etiology and extent (gap-size)) and ATR management (treatment delay, surgical or non-surgical treatment and methods, methods and length of rehabilitation, imaging applied) data. Additionally, the medical status will be monitored throughout the study period for information on injury, treatment and complications if applicable.

#### PROMs

The Achilles Tendon Total Rupture Score (ATRS) is a questionnaire used to measure outcome related to symptoms and physical activity after treatment in patients with an ATR [41]. It consists of ten questions each concerning ten points. This instrument is a valid and reliable method of measuring outcome in ATR patients [41]. It is a self-administered instrument with high clinical applicability, and the score can be used to measure the outcome related to symptoms and physical activity, after treatment in patients with a ATR. In this study the Dutch version will be used (ATRS-NL) which is found to be valid and reliable [42]. In the Dutch version the maximum score (=maximum disability) =100. This measurement will serve as the primary outcome in the analyses.

The Dutch version of the EQ-5D-5L is the most commonly used generic questionnaire to measure quality of life. It encompasses physical, mental, emotional and social functioning. This questionnaire is used to make decisions in cost-effectiveness analyses [43]. The EQ-5D-5 L is scored on a 5-point Likert scale. The result score is a 5-number index score, reflecting the individual’s health-profile. This score can be converted to a total score between 0 (death) and 1 (completely healthy).

The OSTRC Overuse Injury Questionnaire is a 5-question tool used to estimate the burden of injury with respect to RTS, specifically with respect to knee, shoulder or lower back injury. This questionnaire has been found valid [44]. We have modified the Dutch version of this questionnaire to pertain to Achilles tendon injury. This questionnaire will be used to determine the effect of injury on the RTS.

The Injury Psychological Readiness Return to Sport (I-PRRS) Questionnaire assesses an athlete’s psychological readiness to RTS after injury [45]. The I-PRRS is a valid and reliable tool for measuring psychological readiness to RTS. The questionnaire consists of six items that are each scored on a 100-point scale. The total score ranges from 0 to 60 and consists of the sum the six items, divided by 10. A higher score implies greater confidence to RTS. We translated the I-PRRS into Dutch following international guidelines [46, 47].

The Tampa Scale of Kinesiophobia (TSK) measures fear of re-injury due to movement and physical activity. It contains 17 items scored on a four-point Likert scale regarding the subjective experience of the injury and physical activity. The sum of the items results in a score between 17 and 68, where 68 indicates a high level of fear [48]. For this study, the modified Dutch version, adapted for tendon injuries will be used [49]. This questionnaire has been validated in Dutch for measurement of fear of movement/reininjury, although not in an ATR population [49].

A questionnaire concerning expectations, motivation and satisfaction with regard to RTS was constructed. This questionnaire contains 15 questions divided over three dimensions: Expectations (9 questions), Motivation (3 questions) and Satisfaction (3 questions) based on the questionnaire by Sonneson & Ardern (2016) [50]. The questionnaire was designed specifically for use in this study.

A questionnaire concerning reasons for not RTS will be administered. The questionnaire consists of a ranking scale used in prior psychosocial RTS studies after Anterior Cruciate Ligament (ACL) injury [51]. The scale has been translated to Dutch and we modified it to pertain to Achilles tendon injury. The scale was based on the previous reported data on RTS after ACL injury [51]. Patients who reported that they had not returned to their preinjury activity were asked to rank the following reasons for not returning from most important to least important: ‘poor tendon function’, ‘do not trust the tendon, ‘fear getting a new injury’, ‘team or training has changed’, ‘family commitments’, ‘work commitments’ and ‘other reasons’.

#### Physical tests

**Fig. 1:**
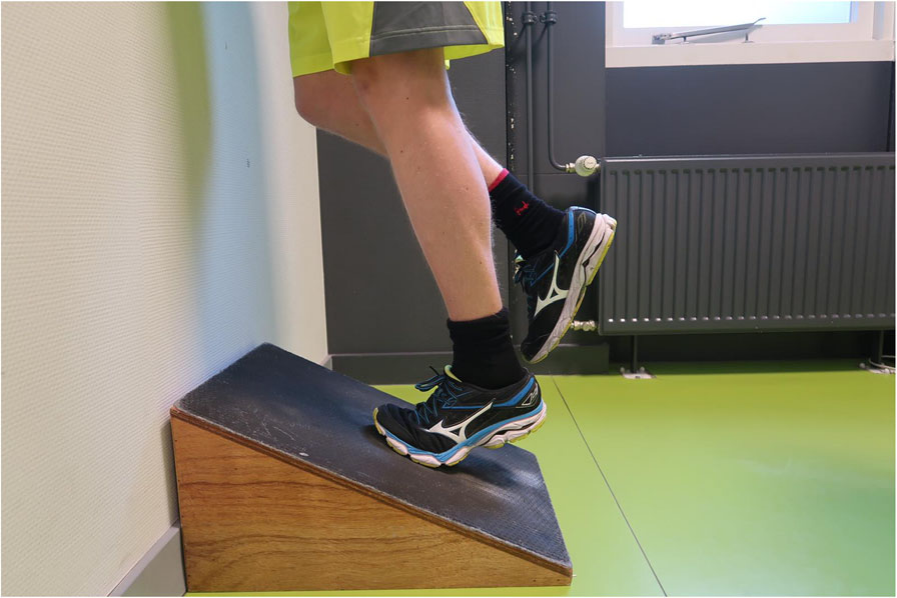
Heel-rise test

Endurance will be assessed with the single leg heel-rise test. This test is a reliable measure of endurance in patients after ATR [52]. With this test patients are instructed to stand on one foot on a 60 degree incline board and perform as many heel-rises as possible. Figure 1 shows the heel-rise test setup. Patients are allowed to have 2 fingertips per hand against the wall for balance, and will perform the rises at a rate of 30 heel rises per minute as guided by a metronome. For each heel-rise they are instructed to go as high as possible and then lower the heel to the starting position. The test will be terminated when the patients stop, cannot maintain the frequency, or cannot perform a proper heel rise [53]. The heel-rise count of the injured foot will be compared to the contralateral (uninjured) foot. The outcome is calculated as the percentage of the heel-rise test count of the ATR-affected side compared to the contralateral (uninjured) limb. The unaffected side is evaluated first.

The tendon length of both the injured and uninjured Achilles tendon will be measured by determining the resting tension on the ankle (the resting plantarflexion with the patients foot prone on an examination table with the feet hanging over the edge). The degree of plantarflexion represents the tendon length [54]. Resting plantarflexion will be measured by using goniometer placed along the lateral border of the foot as described by Ecker et al. [54], this method has been described previously and determined to correlate to muscle strength. The outcome concerns the difference in resting prone plantarflexion between the injured and contralateral foot.

Maximum standing dorsiflexion range of motion (ROM) will be measured with a goniometer with the patient standing. This method has been shown to have good reliability [53, 55]. Care will be taken to place the foot in a subtalarneutral position. The proximal arm of the goniometer will be aligned with the midline of the fibula, the fulcrum with the lateral malleolus, and the distal arm parallel to the fifth metatarsal. The ROM will be compared to the contralateral (healthy) foot.

The entire lower extremity will be tested with the single leg hop for distance. In this test the patient is instructed to stand on one leg and to hop once as far forward as possible, landing only on the same leg. The single leg hop is a reliable test for the function of the lower extremity [56]. The distance from the tip of the patient’s great toe at the starting position to the tip of the patient’s great toe in the landing position is recorded with a tape measure. This test will only be performed at 12 months post-injury. Each leg will be tested three times, and the mean distance hopped over the three repetitions is used for the analysis. The unaffected side is evaluated first.

#### Imaging

**Fig. 2:**
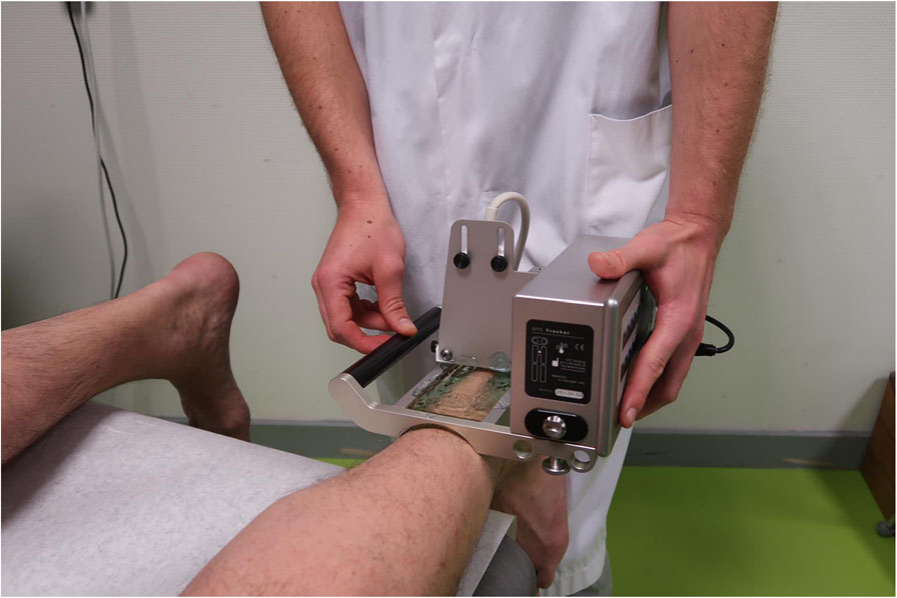
UTC device (probe and tracker) [59]

Ultrasound Tissue Characterization (UTC) is a novel device that can tomographically visualise and accurately quantify tendon structure in three planes. The device has a 7–10 mHZ transducer that moves automatically over tendons and makes transverse recordings over regular intervals of 0.2 mm (Fig. 2) [57]. Operator-dependent variables like transducer tilt, angle, gain and depth are standardized in this scanning method. Tendon structure is quantified via the analysis of the Achilles tendon based on echo-type stability. UTC quantifies tendon structure in four distinct types (I-IV) depending on the amount of fibrillar disorganization and tendon integrity. Echo-type I is the most stable echo pattern and echo-type IV is the least stable echo pattern [57]. The entire tendon will be analyzed. The outcome consists of the percentage of the four different echo-types. This quantification of tendon structure provides the possibility to monitor subtle changes [57]. The UTC has not been used ATR in patients, but has shown potential as a monitoring device in the evaluation of tendinopathy patients and assessing the quality of (Achilles) tendon structure [57].

#### Economic questionnaires

For productivity costs the the institute for Medical Technology Assessment (iMTA) Productivity Cost Questionnaire (iPCQ) will be administered. The iPCQ is a specially constructed Dutch questionnaire designed to measure the indirect costs associated with treatment. It contains three modules (absenteism, presenteeism and productivity losses related to and unpaid work) designed to measure all of the indirect costs associated with medical treatment [58].

For direct medical costs the iMTA Medical Consumption Questionnaire (iMCQ) will be administered. The iMCQ is a specially constructed Dutch questionnaire designed to measure the direct costs associated with treatment. The questions concern ambulance and emerency room use, visits to general practice and the hospital and use of physiotherapty and alternative (homeopathy, acupuncture, e.g.) care. The iMTA costing tool handbook will be used to to collect reference value for treatment costs in the Netherlands.

### Statistical analysis

IBM SPSS Statistics for Windows software (Version 23.0, Armonk, NY: IBM Corp.) will be used for all statistical data analyses. Descriptive statistics (frequencies, means and standard deviations, etc.) will be used to describe all data. Spearman’s correlation coefficients will be used to describe association between data. Statistical significance is defined as *P* < 0.05.

The following are the planned statistical analyses *per aim*:

- *Primary aim:*
  - Generalized estimating equations (GEEs) will be conducted to assess the outcomes over time: 3, 6, and 12 months.
- *Secondary aim:*
  - Linear regression analyses will be performed to assess factors predictive of Achilles tendon function. First, univariate analysis will be performed as a hypothesis-generating analysis model with ATRS-NL score (0–100) as dependent variable and a predicting factor as independent factor. Factors showing a relation with the outcome measure with a *p*-value < 0.20 will be included in a multiple linear regression analysis. Additionally, binary logistic regression will be used to determine the factors associated with returning to the pre-injury sport or recreational activity. Return to the pre-injury activity (yes or no) is the outcome variable.
  - The Incremental Cost-effectiveness Ratio (ICER) (=Δ Cost of treatment/Δ Effect of treatment) associated with each unique treatment modality will be calculated. The ICER is calculated by taking the difference in costs associated with each possible treatment modality and its alternative (e.g. physiotherapy vs. no physiotherapy) and dividing by the difference in effect. Costs are calculated by adding the direct (e.g. cost of treatment from iMCQ questionnaire) and indirect (e.g. loss of work, from iPCQ questionnaire) costs associated with each possible treatment. Effect consists of the EQ-5D-5 L and/or ATRS-NL outcomes. The cost-effectiveness of ATR management options will herewith be determined at 3, 6, and 12 months post injury.

## Discussion

There is an increasing incidence of ATR, and numerous long term impairments are reported. Despite several randomized controlled trials comparing various methods of primary treatment, clinical treatment and rehabilitation guidelines are still lacking. This multicenter prospective cohort study focuses specifically on the rehabilitation phase. This study will contribute to the primary treatment and rehabilitation of ATRs by providing insight into (1) ATR recovery, (2) novel imaging for monitoring recovery, (3) (barriers to) RTS and (4) cost-effectiveness of management. These data will provide a broad understanding of ATR recovery as well as provide data on novel imaging and costs, thereby providing knowledge for clinicians in shared decision making and individualizing ATR management.

## Abbreviations

AAOS: American Academy of Orthopedic Surgeons
ACL: Anterior Cruciate Ligament
ATR: Achilles tendon rupture
ATRS: Achilles Tendon Total Rupture Score
GEE: Generalized estimating equation
ICER: Incremental Cost-effectiveness Ratio
iMCQ: Medical Consumption Questionnaire
iMTA: Institute for Medical Technology Assessment
iPCQ: Productivity Cost Questionnaire
I-PRRS: Injury psychological readiness return to sport scale
MCL: Medical Center Leeuwarden
MHG: Martini Hospital Groningen
OSTRC: Oslo Sport Trauma Research Center
PROMs: Patient-reported outcome measures
ROM: Range of motion
RTS: Return to sport
TSK: Tampa Scale of Kinesiophobia
UMCG: University Medical Center Groningen
UTC: Ultrasound Tissue Characterization
